# *Malus floribunda* Siebold ex Van Houtte Fruit Extract Mitigates Fructose/Streptozotocin Induced Type 2 Diabetes in Rats

**DOI:** 10.3390/ijms27125520

**Published:** 2026-06-18

**Authors:** Muhammed Yayla, Damla Binnetoglu, Erdem Toktay, Huseyin Fatih Gul, Sakir Akgun, Sefa Gozcu, Ugur Ermis, Bengul Ozdemir Sarikaya, Merve Dolunay Uyanik

**Affiliations:** 1 Department of Pharmacology, Faculty of Medicine, Selcuk University, Konya 42100, Turkey; 2 Department of Pharmacology, Faculty of Medicine, Kafkas University, Kars 36100, Turkey; damla.binnetoglu@kafkas.edu.tr (D.B.); ugur.ermis@kafkas.edu.tr (U.E.); 3 Department of Histology and Embryology, Faculty of Medicine, Kafkas University, Kars 36100, Turkey; erdem.toktay@kafkas.edu.tr (E.T.); bengul.ozdemir@kafkas.edu.tr (B.O.S.); 4 Department of Biochemistry, Faculty of Medicine, Kafkas University, Kars 36100, Turkey; huseyin.gul@kafkas.edu.tr; 5 Department of Medical Biology, Faculty of Medicine, Kafkas University, Kars 36100, Turkey; sakir.akgun@kafkas.edu.tr (S.A.); 252516001@ogr.kafkas.edu.tr (M.D.U.); 6 Department of Pharmacognosy, Faculty of Pharmacy, Erzincan Binali Yildirim University, Erzincan 24100, Turkey; sgozcu@erzincan.edu.tr

**Keywords:** diabetes, insulin resistance, *Malus floribunda*, phytotherapy, rat

## Abstract

We aimed to investigate the potential antidiabetic effects of an ethanol extract derived from the fruit of *Malus floribunda* (MF) on insulin resistance, oxidative stress, inflammation, and apoptosis associated with diabetes. In our study, diabetes was induced through the administration of a 10% fructose solution and 40 mg/kg Streptozotocin (STZ). Once diabetes had been induced, metformin (Met) 300 mg/kg and the MF extract (250 mg/kg and 500 mg/kg) were administered orally once daily for 30 days. At the end of the experiment, markers of insulin resistance, oxidative stress, inflammation and apoptosis were evaluated in the serum, muscle and liver tissues of the different groups. The MF extract significantly improved the levels of HOMA-IR, insulin receptor (InR), insulin receptor substrate 1 (IRS-1) and glucose transporter 4 (GLUT4)—key components of peripheral insulin resistance associated with type 2 diabetes. Fructose/streptozotocin induced oxidative stress, inflammation, and apoptosis were mitigated by increasing Nuclear factor erythroid 2-related factor 2 (NRF2) expression, which restored antioxidant levels (Superoxide dismutase (SOD) and Glutathione (GSH)), significantly improved cytokine levels (Tumor necrosis factor alpha (TNF-α) and Interleukin-1 beta (IL-1β)), and downregulated apoptotic proteins (caspase-3 and caspase-9). We demonstrated the antidiabetic effect of MF extract using a fructose/streptozotocin-induced type 2 diabetes model. MF extract shows promise for future use in herbal medicine.

## 1. Introduction

Insulin resistance is defined as an impaired biological response to insulin stimulation in target tissues, primarily the liver, muscle and adipose tissue. It can lead to hyperglycaemia, hypertension, dyslipidaemia, hyperuricaemia, elevated inflammatory markers, endothelial dysfunction and prothrombotic metabolic states. It most commonly presents as type 2 diabetes (T2D) [1]. According to the 2021 International Diabetes Federation (IDF) report, there are currently 537 million people with diabetes worldwide. It is estimated that this figure could rise to approximately 783 million by 2045, representing a 46% increase [2]. The cornerstone of type 2 diabetes management is promoting a healthy lifestyle that includes a balanced diet, regular exercise, and maintaining a healthy body weight. If these changes are insufficient to control blood sugar levels, treatment typically begins with oral medication. Metformin, which helps to prevent insulin resistance, is the first-line therapy. However, metformin can cause side effects such as gastric irritation, cramps, diarrhoea, a metallic taste in the mouth, vitamin B12 deficiency and lactic acidosis. Additionally, vitamin B12 deficiency may lead to anaemia and peripheral neuropathy in the long term [3]. Despite the availability of these treatment options, diabetes still ranks among the top 10 causes of death [2]. For this reason, preclinical and clinical studies are continuously being conducted worldwide to develop new strategies and more effective drug therapies with fewer side effects for the prevention and treatment of diabetes and its associated complications. The *Malus floribunda* is a small tree with dark red fruit and alternate leaves. It is also known as the Japanese flowering apple or the ornamental apple. Its fruit is very small and tastes tart. Kırbağ et al. conducted in vitro analyses of *Malus floribunda* fruit extracts (ethanol) and fruit juices, and the results suggested that they may have antioxidant and antibacterial properties [4]. Çoklar et al. also determined the levels of organic acids, sugars and phenolic compounds in ethanol extracts prepared from the peel, fruit and seeds of *M. floribunda* and demonstrated their antioxidant activity [5]. In his thesis, Zaimoglu investigated the enzyme activities of many different plant species, evaluating the aldose reductase activity of subfractions of an ethanol extract from the fruits of *M. floribunda* and demonstrating that they exhibited approximately 60% enzyme inhibition [6]. Sola et al. evaluated the effect of an ethanol extract derived from *M. floribunda* fruits on the inhibition of alpha-amylase and alpha-glucosidase enzymes, and the results showed that the extract inhibited alpha-glucosidase by over 80% and alpha-amylase by over 15% [7]. Studies have shown that *M. floribunda* fruit contains protocatechuic acid, cinnamic acids, rutin, isorhamnetin-3-glucoside, quercetin, procyanidin, catechin, cyanidin-3-galactoside, anthocyanins, chlorogenic acid, epicatechin, gallic acid, caffeic acid, rutin and morin [5,7,8]. A review of the literature shows that each of these bioactive compounds may possess potent antioxidant, anti-inflammatory and antidiabetic effects. There is insufficient research in the literature on the in vitro and in vivo biological activity of M. floribunda. As it is widely consumed in Turkey and believed by some diabetic patients to be beneficial, evaluating its bioactivity, particularly with regard to diabetes, is of great importance [4]. In our study, we investigated the potential antidiabetic effects of an ethanol extract derived from the fruit of *M. floribunda*. Our objectives were to elucidate its effects on insulin resistance and on the oxidative stress, inflammation and apoptosis associated with diabetes.

## 2. Results and Discussion

### 2.1. Effect of MF Extract on Feed Consumption, Water Intake

**Figure 1:**
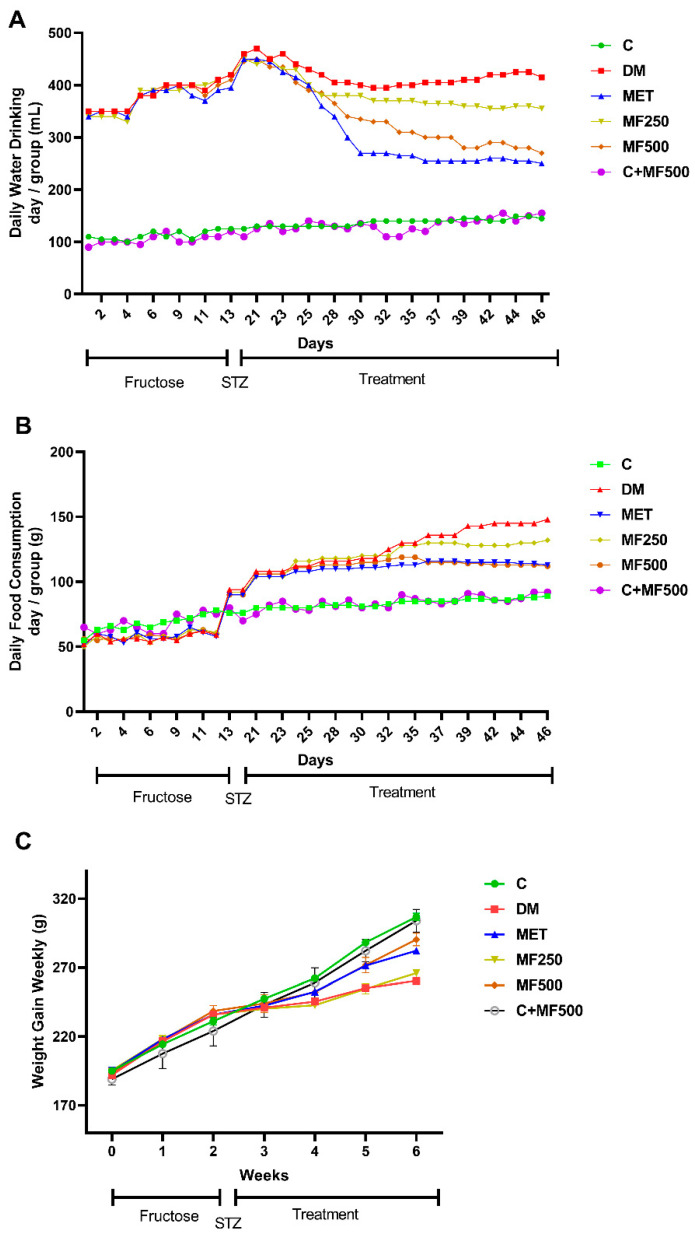
Effect of MF extract on water drinking (**A**), food consumption (**B**), weight gain (**C**), C: Healthy. DM: Diabetes group, MET: Metformin, MF: *Malus floribunda*, STZ: Streptozotocin.

Our study found that feed and water intake increased significantly in groups that developed diabetes compared to the healthy group (Figure 1A,B). By contrast, feed and water intake in the treatment groups receiving Met and MF was lower than in the control group.

### 2.2. Effect of MF Extract on Weight Gain

In our study, weekly weight measurements taken throughout the experiment revealed that body weight decreased in the diabetes and treatment groups compared to the healthy group. However, an increase in body weight was observed in the groups receiving Met and MF500 compared to the diabetes group (Figure 1C).

### 2.3. Effect of MF Extract on FBG, OGTT

**Figure 2:**
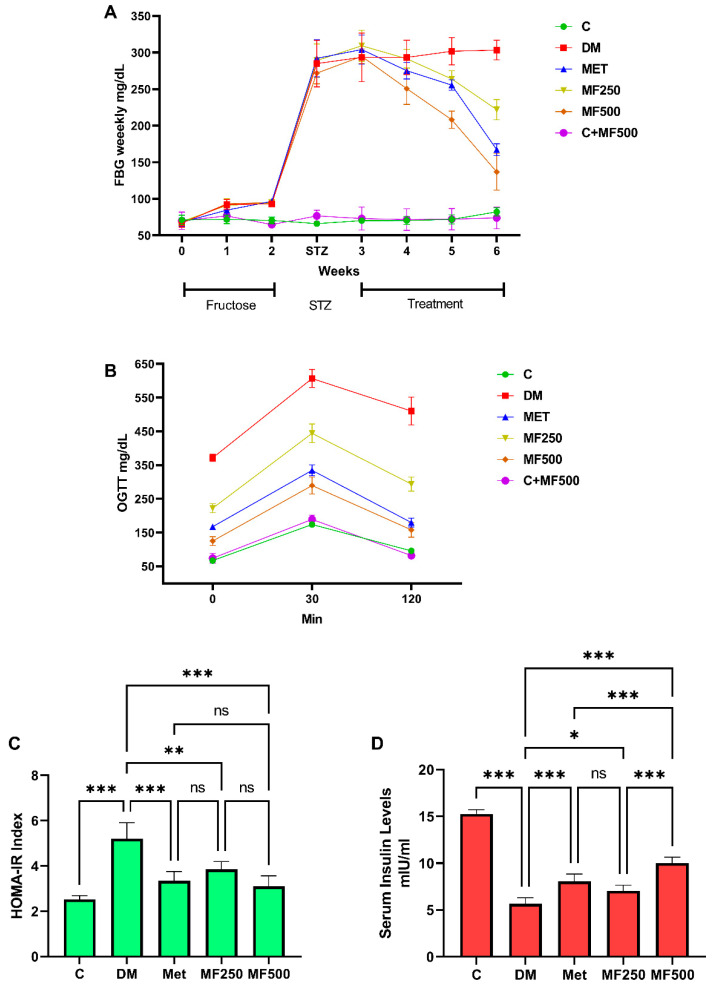
Effect of MF extract on FBG (**A**), OGTT (**B**), HOMA-IR (**C**), Serum insulin levels (**D**). C: Healthy. DM: Diabetes group, Met: Metformin, MF: *Malus floribunda*, STZ: Streptozotocin, ns: non significance, * *p* < 0.05, ** *p* < 0.01, *** *p* < 0.001.

As part of our study, we took weekly blood glucose measurements and administered an orally glucose tolerance test (OGTT) at the end of the experiment. Following STZ administration, the weekly fasting blood glucose (FBG) levels in the diabetic group were found to be significantly higher than in the healthy group. In contrast, FBG levels in the MF and Met groups were found to have decreased significantly compared to the diabetes group (Figure 2A).

Examination of the OGTT results revealed that blood glucose levels in the diabetes group increased significantly at the 30 min time point, with the expected decrease failing to occur at the 120 min time point. In the treatment groups, however, the rise in blood glucose at the 30 min time point was more moderate than in the diabetes group. A significant decrease in blood glucose levels was also observed at the 120 min time point (see Figure 2B).

### 2.4. Effect of MF Extract on Serum Insulin and Insulin Resistance (HOMA-IR)

In our study, we found that fasting insulin levels in the diabetes group were statistically significantly lower than in the healthy group (Figure 2D) (R^2^: 0.9724, *p* < 0.001). However, in the treatment groups, fasting insulin levels were found to have increased significantly compared to the diabetes group (*p* < 0.001 for met group, *p* = 0.0186 for MF250 group, *p* < 0.001 for MF500 group, *p* < 0.05).

Examining insulin resistance (HOMA-IR) data revealed that the HOMA-IR index was significantly higher in the diabetes group (R^2^: 0.8311, *p* < 0.001). In contrast, this value decreased significantly in the MET, MF250 and MF500 treatment groups (Figure 2C) (*p* < 0.001 for met group, *p* = 0.001 for MF250 group, *p* < 0.001 for MF500 group). However, no statistically significant difference was found when comparing the treatment groups regarding this decrease in HOMA-IR levels (*p* > 0.05).

### 2.5. Effect of MF Extract on Serum Lipid Marker

**Figure 3:**
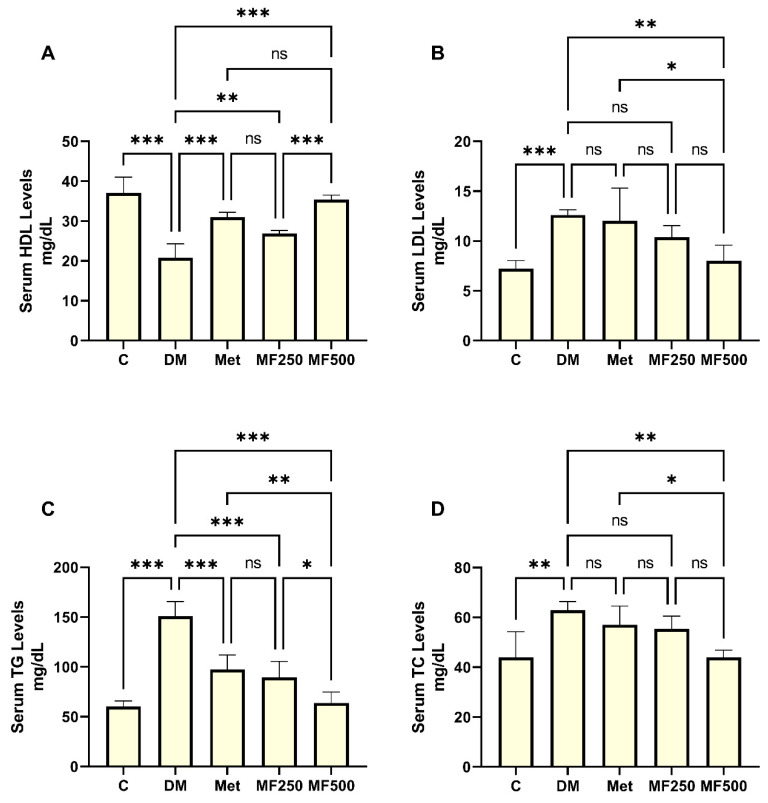
Effect of MF extract serum HDL Levels (**A**), LDL Levels (**B**), TG Levels (**C**), TC Levels (**D**). DM: Diabetes group, Met: Metformin, MF: *Malus floribunda*, ns: non significance, * *p* < 0.05, ** *p* < 0.01, *** *p* < 0.001.

In our study, we found that high density lipoprotein (HDL) levels in the DM group were significantly lower than in the healthy group, while low density lipoprotein (LDL), triglyceride (TG) and total cholesterol (TC) levels were markedly higher (R^2^: 0.8727, for HDL, *p* < 0.001, R^2^: 0.6429, *p* < 0.001 for LDL, R^2^: 0.8890, *p* < 0.001 for TG, R^2^: 0.6232, *p* = 0.002 for TC). A significant improvement in HDL and TG levels was observed in the group treated with MET (*p* < 0.001). The 500 mg/kg dose of MF extract resulted in a statistically significant improvement in all HDL, LDL, TG and TC levels (Figure 3A–D) (*p* = 0.0001, *p* = 0.005, *p* < 0.001, *p* = 0.002, respectively).

### 2.6. Effect of MF Extract on Serum Hepatic and Renal Function Markers

**Figure 4:**
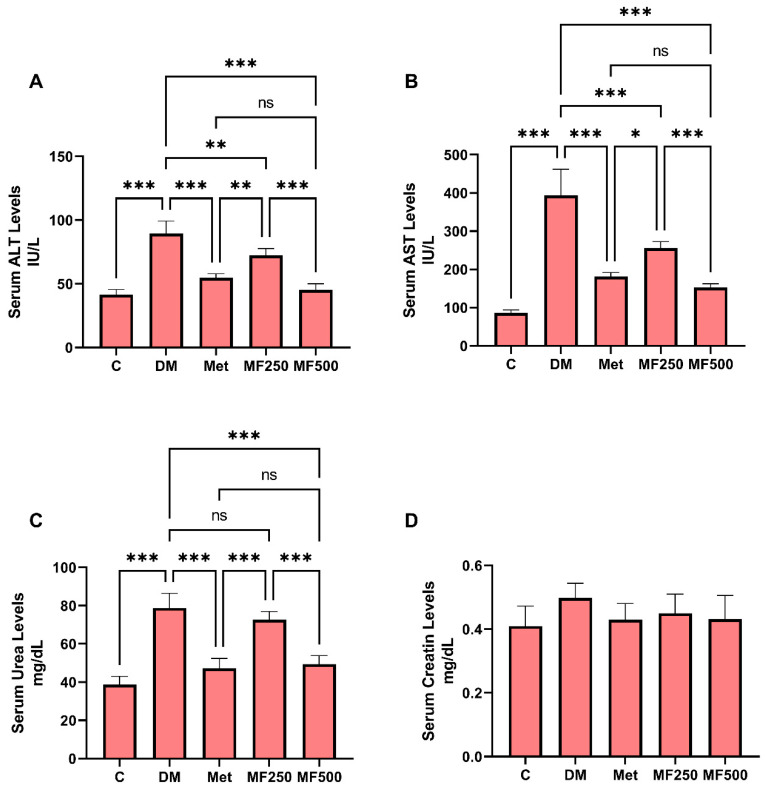
Effect of MF extract serum ALT levels (**A**), AST Levels (**B**), Urea Levels (**C**), Creatine Levels (**D**). DM: Diabetes group, Met: Metformin, MF: *Malus floribunda*, ns: non-significance, * *p* < 0.05, ** *p* < 0.01, *** *p* < 0.001.

In our study, we found that serum alanine aminotransferase (ALT), aspartate aminotransferase (AST) and urea levels were significantly higher in the diabetic groups than in the healthy group (R^2^: 0.9220 for ALT, R^2^: 0.9279 for AST, R^2^: 0.9128 for urea, *p* < 0.001), while creatinine levels did not increase significantly (R^2^: 0.2386 for creatinine, *p* = 0.17). In the treatment groups receiving MET and MF, serum ALT, AST and urea levels improved to a statistically significant degree compared to the diabetes group (Figure 4A–D) (*p* < 0.001, *p* = 0.001, *p* < 0.001, *p* < 0.001, *p* < 0.001, *p* < 0.001, *p* < 0.001, *p* = 0.39, *p* < 0.001, respectively). The group treated with a 500 mg/kg dose of MF extract exhibited a dose-dependent effect (*p* < 0.05).

### 2.7. Effect of MF Extract on Serum and Tissue Oxidative Stress

**Figure 5:**
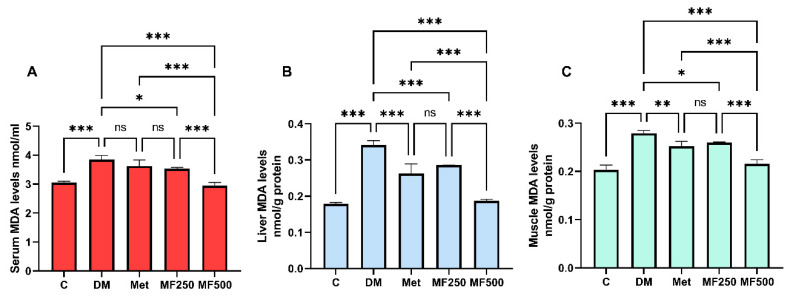
Effect of MF extract serum and tissue oxidative stress markers, serum MDA levels (**A**), Lİver MDA levels (**B**), Muscle MDA levels (**C**). DM: Diabetes group, Met: Metformin, MF: *Malus floribunda*, ns: non significance, * *p* < 0.05, ** *p* < 0.01, *** *p* < 0.001.

In our study, we observed a statistically significant increase in malondialdehyde (MDA) levels in serum, liver and muscle tissue in the diabetes group compared to the healthy group (Figure 5A–C) (R^2^: 0.9064 for serum MDA, R^2^: 0.9647 for liver MDA, R^2^: 0.9458 for muscle MDA, *p* < 0.001). In contrast, significant improvements were observed in liver and muscle MDA levels in the MET-treated group (*p* < 0.001, *p* = 0.002). The MF extract exhibited a dose-dependent effect and provided significant improvements in serum, liver and muscle tissue levels at the 500 mg dose compared to the diabetic group (*p* = 0.03, *p* < 0.001, *p* < 0.001, *p* < 0.001, *p* = 0.03, *p* < 0.001).

**Figure 6:**
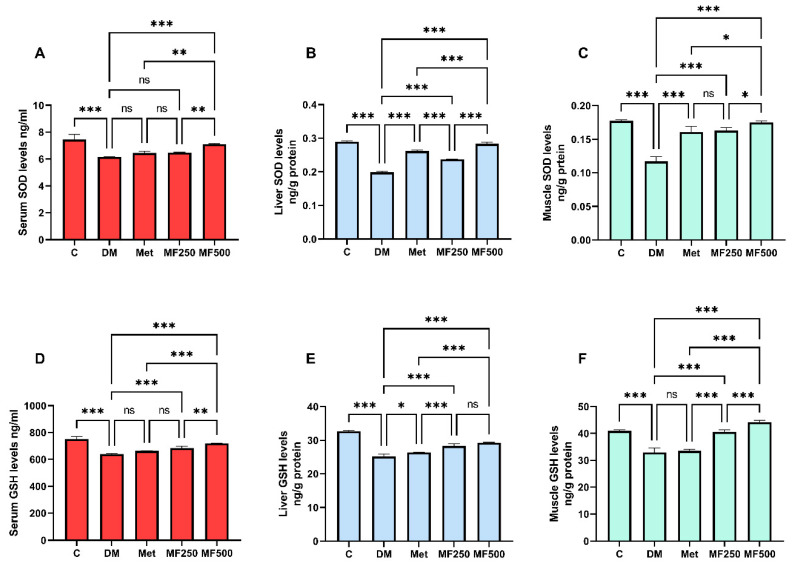
Effect of MF extract serum and tissue oxidative stress markers, serum SOD levels (**A**), liver SOD levels (**B**), Muscle SOD levels (**C**), serum GSH levels (**D**), liver GSH levels (**E**), muscle GSH levels (**F**). DM: Diabetes group, Met: Metformin, MF: *Malus floribunda*, ns: non significance, * *p* < 0.05, ** *p* < 0.1, *** *p* < 0.001.

Examining antioxidant parameters revealed that serum, liver and muscle tissue SOD and GSH levels in the diabetes group were significantly lower than in the healthy group (Figure 6A–F) (R^2^: 0.9033 for serum SOD, R^2^: 0.9919 for liver SOD, R^2^: 0.9569 for muscle SOD, R^2^: 0.9442 for serum GSH, R^2^: 9743 for liver GSH, R^2^: 0.9684 for muscle GSH, *p* < 0.001). In groups treated with MF, a statistically significant increase in SOD and GSH levels was observed in a dose-dependent manner (*p* = 0.14, *p* < 0.001, *p* < 0.001, *p* < 0.001, *p* < 0.001, *p* < 0.001, *p* < 0.001, *p* < 0.001, *p* < 0.001, *p* < 0.001, *p* < 0.001, *p* < 0.001, respectively), with the most pronounced improvement seen at the 500 mg MF dose.

### 2.8. Effect of MF Extract on Serum and Tissue Inflammation

**Figure 7:**
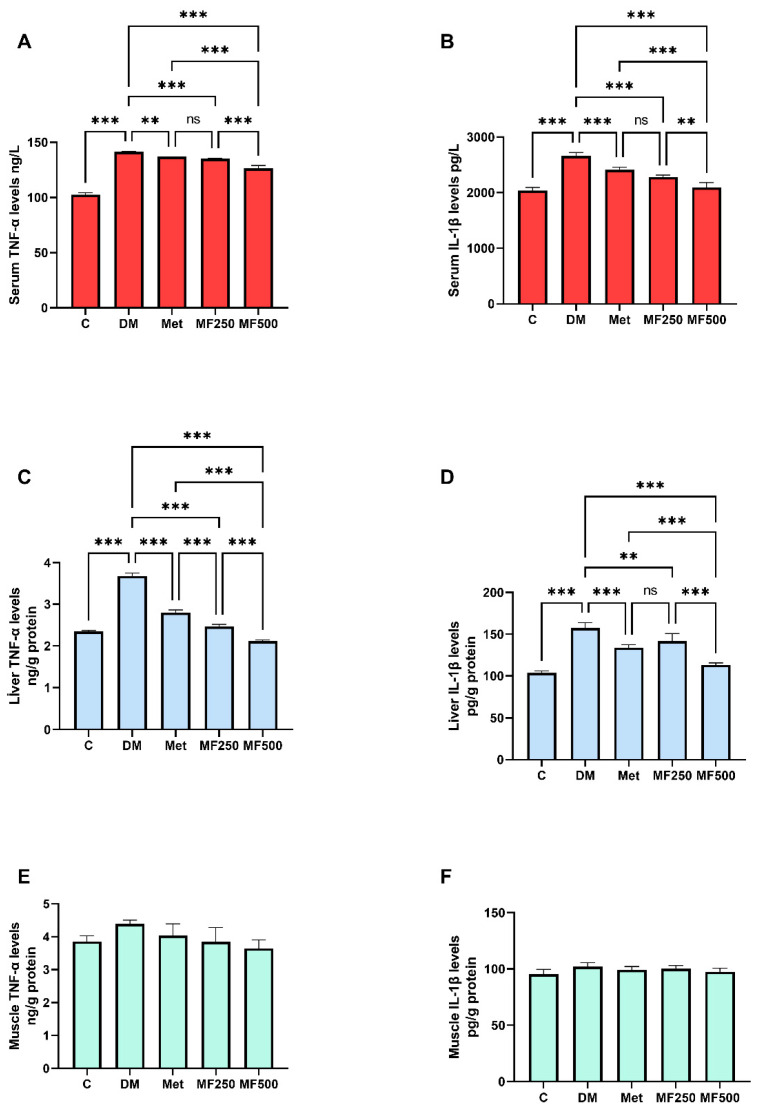
Effect of MF extract serum and tissue inflammatory markers, serum TNF-α levels (**A**), serum IL-1β levels (**B**), liver TNF-α levels (**C**), Liver IL-1β levels (**D**), muscle TNF-α levels (**E**), muscle IL-1β levels (**F**), DM: Diabetes group, Met: Metformin, MF: *Malus floribunda*, ns: non significance, ** *p* < 0.01, *** *p* < 0.001.

Figure 7A–D shows that serum and liver TNF-α and IL-1β levels were significantly higher in the diabetes group than in the healthy group (R^2^: 0.9920 for serum TNF-α, R^2^: 0.9934 for liver TNF-α, R^2^: 0.9510 for serum IL-1β, R^2^: 0.9455 for liver IL-1β, *p* < 0.001). In the MET, MF250 and MF500 groups, serum and liver TNF-α and IL-1β levels were significantly lower than in the diabetes group (*p* = 0.006, *p* < 0.001, *p* < 0.001, *p* < 0.001, *p* < 0.001, *p* < 0.001, *p* < 0.001, *p* < 0.001, *p* < 0.001, *p* < 0.001, *p* = 0.007, *p* < 0.001, respectively). The MF group exhibited a dose-dependent effect (*p* < 0.001). No significant changes in TNF-α and IL-1β levels were observed among the groups in muscle tissue (Figure 7E,F) (R^2^:0.4918 for muscle TNF-α, R^2^:0.3810 for muscle IL-1β, *p* = 0.12, *p* = 0.48, *p* = 0.88, *p* = 0.35, *p* = 0.08, *p* = 0.76, *p* = 0.93, *p* = 0.80, respectively).

**Figure 8:**
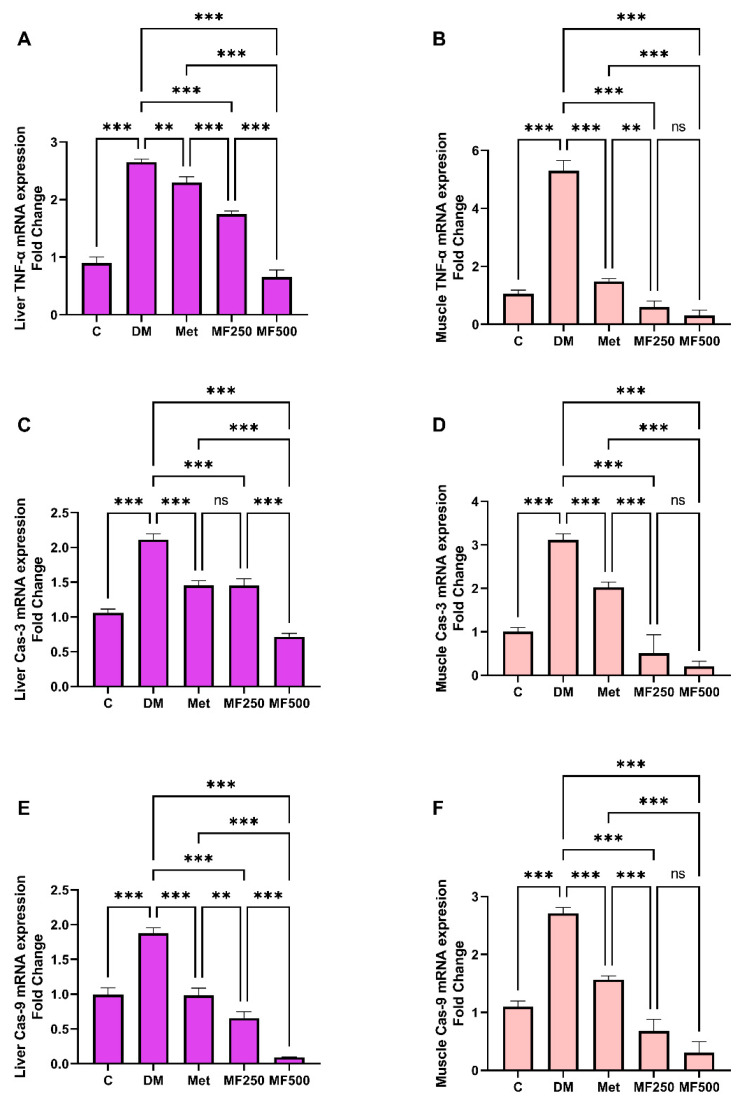
Effect of MF extract tissue inflammatory apoptotic protein gene expressions, liver TNF-α expressions (**A**), muscle TNF-α expressions (**B**), liver Caspase 3 expressions (**C**), muscle caspase 3 expressions (**D**), liver Caspase 9 expressions (**E**), muscle caspase 9 expressions (**F**). DM: Diabetes group, Met: Metformin, MF: *Malus floribunda*, ns: non significance, ** *p* < 0.01, *** *p* < 0.001.

Evaluating TNF-α mRNA expression in the liver revealed that it was significantly upregulated in the diabetes group (*p* < 0.001). In contrast, the MF group significantly downregulated it in a dose-dependent manner (Figure 8A) (R^2^: 0.9912 for liver TNF-α, *p* < 0.001). TNF-α mRNA expression levels in muscle tissue were significantly higher in the diabetes group than in the healthy group (Figure 8B) (R^2^: 0.4918, *p* < 0.001), while they were significantly lower in the groups treated with MET and MF (*p* < 0.001).

### 2.9. Effect of MF Extract on Tissue Apoptosis

Examination of the mRNA expression levels of caspase-9 and caspase-3 in liver tissue revealed a statistically significant upregulation in the diabetic group compared to the healthy group (Figure 8C,E) (*p* < 0.001). In the treatment groups receiving MET and MF, a significant downregulation of these genes was observed. It was noted that the MF extract exhibited a dose-dependent effect on this reduction (R^2^: 0.9842 for liver caspase 3, R^2^: 0.9865 for liver caspase 9, *p* < 0.001). The mRNA expression levels of caspase-3 and -9 in skeletal muscle were also found to be significantly higher in the diabetes group than in the healthy group (Figure 8D,F) (R^2^: 0.9741 for muscle caspase 3, R^2^: 0.9808 for muscle caspase 9, *p* < 0.001). In the groups treated with Met and MF, these levels were significantly downregulated (*p* < 0.001).

### 2.10. Effect of MF Extract on Tissue Histopathology and NRF-2 Levels

#### 2.10.1. Liver Tissue Findings

**Figure 9:**
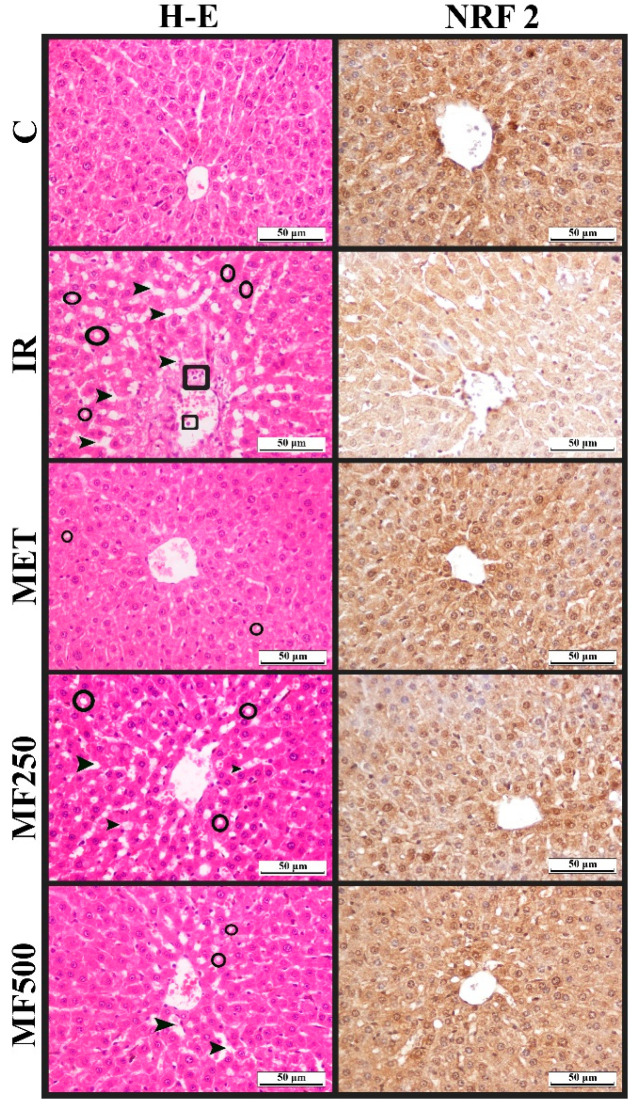
Findings from hematoxylin and eosin staining and immunohistochemical staining of liver tissue (Circle: Sinusoidal dilations; arrow: Lipid deposits; Square: Inflammatory cells).

Histopathological examinations of liver tissue revealed that the classic liver lobules were intact and that the hepatocyte cords were arranged around the central vein in sections from the healthy group. By contrast, the DM group showed severe dilation between hepatocyte cords, intercellular lipid accumulation and inflammatory cells in the sinusoidal spaces. In the group treated with MET, no sinusoidal dilation was evident, though minimal lipid accumulation was noted. In the treatment groups administered MF, a significant reduction in sinusoidal dilation and lipid accumulation was observed in a dose-dependent manner (Figure 9).

Immunohistochemical staining with the NRF-2 antibody in liver tissue revealed mild (1) immunopositivity in the DM group, moderate (2) immunopositivity in the DM + MF 250 group, and severe (3) immunopositivity in the DM + MET, DM + MF 500, and healthy groups (Figure 9 and Table 1).

#### 2.10.2. Muscle Tissue Findings

**Figure 10:**
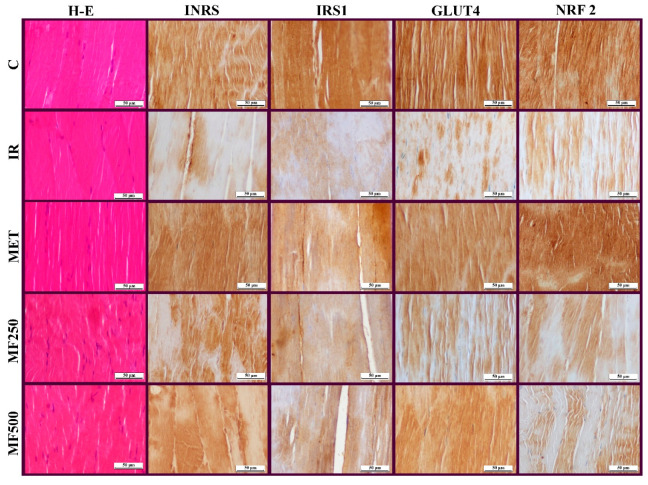
Findings from hematoxylin and eosin staining and immunohistochemical staining in muscle tissue.

Histopathological examinations of muscle tissue revealed no significant differences in pathology between the experimental groups (Figure 10). Immunohistochemical staining using the NRF-2 antibody revealed mild (1+) immunopositivity in the DM group, moderate (2+) in the DM + MF 250 and DM + MF 500 groups, and severe (3+) in the healthy and DM + MET groups (see Figure 10 and Table 1).

### 2.11. Effect of MF Extract on Tissue Insulin Resistance

Immunohistochemical examinations of muscle tissue revealed the following staining results: mild (1) in the DM group, moderate (2) in the DM + MF 250 and DM + MF 500 groups, and severe (3) in the DM + MET and healthy groups (Figure 10 and Table 2).

According to the results of staining with the IRS1 antibody, mild immunopositivity (1) was observed in the DM group, moderate immunopositivity (2) in the DM + MF 250 group, and severe immunopositivity (3) in the healthy group, as well as in the DM + MF 500 and DM + MET groups (Figure 10 and Table 2).

Using the GLUT4 antibody, mild immunopositivity (1) was observed in the DM group, moderate immunopositivity (2) in the DM + MF 250 group, and severe immunopositivity (3) in the healthy, DM + MF 500, and DM + MET groups (Figure 10 and Table 2).

The Japanese apple (*Malus floribunda*) grows in many regions of our country. In Turkey, people with diabetes consume its fruits for their anti-diabetic properties. Additionally, some studies have demonstrated its inhibitory activity against aldose reductase, alpha-amylase and alpha-glucosidase in vitro [6,7]. Recent studies have examined its antioxidant and antimicrobial effects [4,5,7,8]. The fruit is rich in bioactive vitamins and phenolic compounds. In our study, LC-MS/MS analysis of the *Malus floribunda* fruit extract revealed a rich profile of phenolics and flavonoids, with isoquercitrin (0.476 mg/g), chlorogenic acid (0.340 mg/g), catechin (0.285 mg/g), and quercetin-3-D-xyloside (0.161 mg/g) identified as the major secondary metabolites. The profound antidiabetic, antioxidant, and anti-inflammatory effects observed in the MF-treated groups can be attributed to the synergistic action of these major compounds [9].

Based on these studies, it was determined that, in the diabetes group, food, water intake and fasting blood glucose levels increased and could not be lowered during the OGTT test, and the HOMA-IR index increased [10]. MF extract improved feed, water intake, fasting blood glucose levels, OGTT and HOMA-IR index. This suggests that MF extract can improve the insulin resistance associated with diabetes. The most fundamental literature supporting the antidiabetic activity of MF extract in vitro is the demonstration that it can inhibit the enzymes alpha-amylase, alpha-glucosidase and aldose reductase [6,7]. These data may serve as primary evidence to support the finding of our study that MF extract improves diabetic parameters, particularly fasting blood sugar levels.

In the T2DM model, high fructose intake dramatically increases levels of plasma free fatty acids (FFA), TG and LDL by enhancing de novo lipogenesis in the liver [11,12,13]. In our study, we observed a significant decrease in HDL levels in the diabetic group compared to the healthy group, while significant increases in LDL, TG and TC levels were observed. MF extract produced a significant, dose-dependent improvement in HDL, LDL, TG and TC levels.

Hyperlipidaemia and STZ-induced hyperglycaemia lead to ectopic lipid accumulation in the liver and skeletal muscle, the body’s primary metabolic organs, and directly disrupt the insulin signalling mechanisms responsible for cellular glucose uptake [14]. Diabetes-related lipotoxic and hyperglycaemic stress in skeletal muscle disrupts IRS-1 and phosphatidylinositol 3-kinase (PI3K)/AKT signalling pathways [15]. In skeletal muscle in particular, fructose- and STZ-mediated damage inhibits the PI3K/AKT pathway, thereby preventing the translocation of GLUT4 molecules to the plasma membrane and exacerbating systemic insulin resistance [16,17].

Similarly, our study found that the expression of InR, IRS-1 and Glut4 in the muscle tissue of the diabetic group was significantly lower than in the healthy group. Roy et al. demonstrated in their study that the expression of InR and Glut4 in muscle tissue associated with type 2 diabetes was significantly reduced [18]. In the MF group, the expression of InR, IRS-1 and Glut4 improved in a dose-dependent manner in muscle tissue. Chlorogenic acid and isoquercitrin have been widely reported to enhance GLUT4 translocation and improve peripheral insulin sensitivity via the activation of AMP-activated protein kinase (AMPK) and IRS-1 pathways, which strongly aligns with our immunohistochemical findings showing significant upregulation of InR, IRS1, and GLUT4 in skeletal muscle [19,20].

Diabetes-related hyperglycaemia and lipotoxicity lead to the production of reactive oxygen species (ROS) at destructive levels by overloading the mitochondrial electron transport chain in the liver and skeletal muscle [21]. Excessive ROS accumulation triggers lipid peroxidation in cell membranes, thereby increasing levels of toxic end products such as MDA [11,22]. This increase in oxidative stress leads to tissue dysfunction by severely depleting the levels and function of key endogenous antioxidant defences, such as SOD, catalase (CAT) and GSH, which are responsible for maintaining cellular redox balance in the liver and skeletal muscle [23,24].

In our study, we observed higher levels of MDA in the serum, liver and muscle tissue of the diabetic group. This indicates increased oxidative stress in this group. At the same time, we observed a significant decrease in the levels of SOD and GSH, which are important components of the antioxidant defence system. Previous studies have shown that MDA levels increase in serum, liver and muscle tissue in type 2 diabetes, while SOD and GSH levels decrease [18,25,26]. In our study, MF extract produced a significant, dose-dependent improvement in MDA levels in serum, liver and muscle tissue, while also resulting in a significant increase in SOD and GSH levels. Cotelli et al. demonstrated the antioxidant capacity of MF extract through in vitro analysis [8]. Following these analyses, Kırbağ et al. noted that MF extract may exhibit antioxidant activity [4]. In another study, Çoklar et al. demonstrated the antioxidant capacity of MF extract [5]. These findings suggest that MF extract, which is known for its in vitro antioxidant activity, can prevent the oxidative damage associated with diabetes.

Under normal physiological conditions, the transcription factor NRF2 dissociates from its cytoplasmic inhibitor Keap1 upon exposure to oxidative stress. NRF2 then translocates to the nucleus and induces the synthesis of phase II antioxidant genes, such as HO-1, SOD and CAT [24]. However, under severe diabetic conditions, the nuclear translocation and cellular defence activity of NRF2 in the liver and skeletal muscle are paradoxically suppressed [27,28]. This loss of NRF2 pathway function accelerates steatosis in the liver while exacerbating oxidative damage and atrophy in muscle [24,29].

In our study, analyses of liver and muscle tissue revealed a significant decrease in NRF2 expression in the diabetic group. Previous studies have also demonstrated a significant decrease in NRF2 levels associated with diabetes [21,29,30,31]. The MF extract significantly increased NRF2 levels in a dose-dependent manner, thereby stimulating the synthesis of antioxidant genes. This indicates that the MF extract may stimulate the expression of antioxidant genes at the genetic level.

In T2DM, glucolipotoxicity and oxidative stress not only damage cells directly, but also strongly trigger the activation of nuclear factor-kappa B (NF-κB) and c-Jun N-terminal kinase (JNK), which are master switches of pro-inflammatory signalling pathways [12,15]. The NF-κB complex, activated in the liver and muscle, leads to the overexpression of pro-inflammatory cytokines such as TNF-α, IL-6 and IL-1β [32,33]. Conversely, JNK pathway activation acts as a key mediator in the pathogenesis of insulin resistance by phosphorylating the inhibitory domains of IRS-1, thereby inducing tissue damage [15,26]. It has been demonstrated that NRF2 activation successfully suppresses the NF-κB and JNK cascades by reducing cellular ROS levels, thereby breaking the inflammation-insulin resistance cycle in the liver and muscle [24,34].

In our study, serum and liver tissue levels of TNF-α and IL-1β were significantly higher in the diabetic group. Histological analysis revealed the presence of inflammatory cells in the sinusoidal spaces of liver tissue. However, although numerical differences in TNF-α and IL-1β levels were observed in muscle tissue, these were not statistically significant. Furthermore, no inflammatory findings were evident in the histological analysis. At the gene expression level, TNF-α mRNA expression was found to be significantly increased in both liver and muscle tissue. A review of the literature shows that inflammation increases with diabetes and that levels of cytokines such as TNF-α and IL-1β are significantly elevated [12,24,26]. In groups treated with MF extract, TNF-α and IL-1β levels decreased significantly in a dose-dependent manner. This suggests that MF extract may prevent inflammation associated with diabetes. While there are currently no studies supporting the anti-inflammatory effects of MF extract, the strong anti-inflammatory activity of its bioactive components suggests that it may prevent diabetes-associated inflammation [35,36,37,38,39]. Furthermore, quercetin derivatives and catechins are potent NRF2 activators [40,41]. The marked increase in NRF2 tissue expression and the subsequent restoration of endogenous antioxidant enzymes (SOD and GSH) in our study confirm that these major flavonoids successfully mitigate diabetes-induced oxidative stress, thereby suppressing the TNF-α/Caspase-mediated apoptotic pathways [42,43]. Consequently, rather than a single component, the complex phytochemical profile dominated by these key polyphenols constitutes the molecular basis of the therapeutic potential of *M. floribunda*.

In T2DM, uncontrolled chronic hyperglycaemia and lipotoxicity, combined with endoplasmic reticulum stress and an inflammatory response in the liver and skeletal muscle, drive cells into an intrinsic (mitochondrial) apoptotic process [43]. This highly diabetogenic environment suppresses the expression of the anti-apoptotic Bcl-2 protein, which supports cell survival, while causing a sharp increase in levels of the pro-apoptotic Bax protein and cytochrome c release, as well as activating Caspase-3, the primary executor of apoptosis [21,23,28,30]. Activation of the apoptosis cascade leads not only to the permanent destruction of pancreatic β-cell mass, but also to accelerated skeletal muscle atrophy and progressive liver dysfunction, rendering the condition irreversible [34,44].

Furthermore, ensuring the chemical reproducibility and batch-to-batch consistency of herbal extracts is a critical prerequisite for their development as standardized therapeutic agents. In this study, the quantitative LC-MS/MS profiling successfully established specific chemical markers, primarily isoquercitrin and chlorogenic acid, which serve as the baseline for the internal standardization of the *Malus floribunda* fruit extract. For future pilot-scale or commercial-scale translations of this research, preserving these verified quantitative thresholds (e.g., maintaining isoquercitrin levels at approximately 0.47 mg/g) will be mandatory to guarantee reproducible pharmacological efficacy and consumer safety across different production batches.

Importantly, the repeatability of the extraction process was comprehensively confirmed through both quantitative and qualitative evaluations. Quantitative analysis demonstrated that the extraction yield was highly consistent among independent batches, with a yield of 28.0 ± 0.8%. In addition, standardized LC–MS/MS profiling verified that the principal bioactive markers, particularly isoquercitrin and chlorogenic acid, exhibited stable chromatographic behavior and consistent quantitative distributions across different extraction runs. This combined validation strategy minimizes the intrinsic variability associated with complex herbal matrices and ensures that the pharmacological effects observed in the MF-treated groups are reproducible and directly associated with a chemically standardized phytochemical profile.

Finally, our study analysed apoptosis data related to diabetes and observed that the caspase-3 and caspase-9 genes were significantly upregulated in muscle and liver tissues in the diabetes group. A review of the current literature indicates that the expression of apoptotic proteins increases in muscle and liver tissues as a result of diabetes, whereas it is downregulated in treatment groups [12,21,43,44]. In our study, the MF group significantly down-regulated the gene expression of apoptotic proteins in a dose-dependent manner. This suggests that the MF extract may exhibit anti-apoptotic activity. As discussed in our content analysis results, the extract contains numerous bioactive compounds, primarily quercetin, shikimic acid, chlorogenic acid and rutin. Previous studies have demonstrated the anti-apoptotic properties of these molecules [45,46,47,48]. Therefore, our study supports the ability of MF extract to prevent diabetes-associated apoptosis.

Importantly, the novelty of the present study extends beyond the evaluation of a plant extract in an experimental diabetes model. To the best of our knowledge, this is the first study to identify and accurately quantify protocatechuic acid, vanillin, trans-ferulic acid, and protocatechuic acid ethyl ester in *M. floribunda* fruit. The discovery of these previously unreported constituents substantially expands the phytochemical knowledge of this species and provides a new basis for understanding its biological activity [7,8].

In literature, protocatechuic acid has been shown to exert robust insulin-mimetic effects by directly upregulating GLUT4 translocation and glucose uptake in peripheral tissues via the activation of metabolic pathways [49]. Similarly, trans-ferulic acid is well-documented to restore the compromised IRS-1 signaling cascade, improve glucose homeostasis, and alleviate hepatic glucolipotoxicity in high-fructose dietary models [50]. Furthermore, the presence of vanillin provides a strong biochemical explanation for the extract’s profound antioxidant and anti-apoptotic performance; vanillin is known to suppress NF-κB-mediated inflammatory responses and caspase-dependent apoptotic pathways [51].

Furthermore, this study is the first to demonstrate the therapeutic relevance of this phytochemical composition in this study. By linking the newly characterized phenolic profile with improvements in glycemic control, lipid metabolism, oxidative stress, and peripheral insulin resistance, the present work establishes a mechanistic connection between the chemical composition of *M. floribunda* fruit and its antidiabetic effects.

## 3. Materials and Methods

### 3.1. Preparation of the Plant Extract

In our study, sufficient quantities of the fruits of *Malus floribunda* Siebold ex. van houtte (Rosaceae)—a plant traditionally used as an antidiabetic agent—were collected in September from Nigde/Merkez. The fruits were dried in a shaded area, protected from moisture, with adequate air circulation. The plant was identified by Assoc. Prof. Dr. Mustafa Korkmaz, and herbarium specimens are preserved at the Erzincan Binali Yildirim University Herbarium (EBYU). The preparation of the plant extract for this study carried out at the Central Research Laboratory of Erzincan Binali Yildirim University. Based on this study, the dried and powdered plant material was weighed at 2000 g and extracted with 5 L (*w*/*v*) of 75% ethanol under reflux. The solvents were removed using an evaporator. This process was repeated three times. After concentrating to dryness, 560 g of ethanolic extract was obtained, representing a final extraction yield of 28.0 ± 0.8% (*w*/*w*) thereby confirming the quantitative repeatability of the method.

### 3.2. LC-ESI-MS/MS Conditions

Quantitative determination of phytochemicals was carried out using an Agilent 1260 Infinity II LC system coupled to an Agilent 6460 Triple Quadrupole Mass Spectrometer (Agilent, Santa Clara, CA, USA). Chromatographic separation was achieved on an Agilent Poroshell 120 EC-C18 column (100 × 3.0 mm, 2.7 μm) maintained at 40 °C. The mobile phase consisted of (A) water containing 5 mM ammonium formate and (B) acetonitrile supplemented with 0.1% formic acid. A gradient elution program was applied as follows: 3% B (0–3 min), 50% B (4–12 min), 90% B (13–21 min), and re-equilibration to 3% B (22–25 min). The flow rate was set at 0.5 mL/min, with a total run time of 25 min. Mass spectrometric detection was performed in both positive and negative ionization modes using multiple reaction monitoring (MRM). Transitions from precursor to product ions were selected based on MS/MS spectra, and collision energies were optimized to ensure efficient fragmentation and transmission of product ions. Instrumental conditions included a drying gas (N_2_) flow of 15 L/min, nebulizing gas (N_2_) flow of 11 L/min, capillary voltage of 4000 V, gas temperature of 350 °C, heat block temperature of 400 °C, and interface temperature of 350 °C. Data acquisition and processing were performed using Agilent MassHunter software B.09.00. The analytical method was validated by evaluating the limit of detection (LOD), limit of quantification (LOQ), and linearity. Validation data, including retention time (RT), LOD, LOQ, and coefficient of determination (R^2^) for 39 phenolic acids and flavonoids, are summarized in Table 3 and Table S1. The high R^2^ values (>0.99) indicate excellent linearity and confirm the robustness and accuracy of the method.

For sample preparation, 50 mg of the extract was dissolved in 1 mL of methanol and mixed with 1 mL of n-hexane. The mixture was vortexed at 4 °C for 2 min and centrifuged at 9000 rpm for 10 min. The upper hexane layer was discarded, and the methanolic phase was diluted with distilled water (1:9, *v*/*v*). The resulting solution was filtered through a 0.45 μm syringe filter and injected into the system via an autosampler.

### 3.3. Laboratory Animals

Male albino Wistar rats weighing 200–250 g were used in the study. The study consisted of 6 groups. A total of 48 rats were used, with 8 rats in each group. The study was conducted with the approval of the ethics committee of Kafkas University (KAÜ-HADYEK/2024-11). The animals were housed at 22 ± 2 °C, 50–60% humidity, and a 12/12 h light/dark cycle and were fed standard rat chow and water ad libitum.

### 3.4. Fructose/STZ-Induced Insulin Resistance

The animals were randomly divided into groups based on body weight. The drinking water of all groups, except the healthy control group, was adjusted to contain 10% fructose (99.5%fructose, Takita, Turkey) for 15 days. Daily water and food intake and weekly weight and fasting blood sugar levels were monitored and recorded during the experiment. On day 15, the fructose-containing drinking water was discontinued and the animals were given normal drinking water again. Diabetes was induced by administering a single intraperitoneal dose of streptozotocin (BioShop, Burlington, ON, Canada) dissolved in a citric acid and sodium citrate buffer at 0.1 M, pH 4.5, at a dose of 40 mg/kg body weight to rats that had been fasted for approximately 12 h [12,44].

As STZ administration leads to intense insulin secretion from the pancreas due to beta cell destruction, there is an increased risk of hypoglycemia, which can acutely increase mortality in animals. For this reason, a 5% dextrose solution was added to the rats’ drinking water for 24 h. Three to seven days after STZ administration, the rats were fasted for 12 h. Fasting blood glucose levels were measured by evaluating blood glucose levels using a glucometer with blood samples taken from the tail veins. Rats with glucose levels above 250 mg/dL were classified as diabetic and included in the experiment. The extracts were then administered orally via gavage at doses of 250 mg/kg and 500 mg/kg once daily for 30 days. The reference drug metformin (Ali Raif İlaç, İstanbul, Turkey) was then administered orally via gavage at a dose of 300 mg/kg once daily for 30 days. Throughout the experiment, the rats’ drinking water and feed were monitored daily, as were their body weight and blood glucose levels, which were checked at 7-day intervals. The experiment concluded on the 30th day following extract administration. Blood was collected intracardially from the anaesthetized rats. Muscle and liver tissues were excised and stored in a 10% formaldehyde solution for histological analysis and in a freezer at −80 °C for biochemical and molecular analysis. The biochemical, molecular, and histological analyses in this study were conducted in a double-blind manner.

### 3.5. Groups

1. Control (C);
2. Diabetes group (DM) (10% fructose/40 mg/kg STZ) [12,44];
3. DM + Metformin (Met) 300 mg/kg oral [52];
4. DM + *M. floribunda* extract (MF) 250 mg/kg oral;
5. DM + MF 500 mg/kg oral;
6. C + MF 500 mg/kg oral

### 3.6. OGTT Test

At the end of the experiment, the rats were fasted overnight prior to the OGTT test. They were then administered 2 g/kg of glucose orally for the OGTT. Blood glucose levels in the tail vein were measured at 0, 30, and 120 min after glucose administration using a blood glucose meter and test strips [53].

### 3.7. HOMA-IR Index Calculation

The OGTT and serum insulin levels at the end of the experiment were used to calculate insulin resistance using the HOMA-IR formula [HOMA-IR = Insulin (µIU/mL) × FBG (mg/dL)/405] [53,54].

### 3.8. Biochemical Processes

#### 3.8.1. Blood Sample Collection and Preparation

Blood samples of 5 mL were drawn into biochemistry tubes with gel, followed by centrifugation at 3000 rpm for 20 min at room temperature to obtain a clear serum sample. The separated serum samples were aliquoted into Eppendorf tubes and stored at −80 °C until quantitative analyses were conducted.

#### 3.8.2. Preparation of Liver-Muscle Tissues Homogenates

The homogenization of liver and muscle tissues was performed to obtain high-quality supernatants for biochemical analysis. Briefly, the harvested tissues were immediately rinsed in ice-cold phosphate-buffered saline (PBS, 0.01 M; pH 7.4) to remove excess blood and contaminants. For the liver tissue, which is relatively friable, the samples were weighed and diluted in a 1:10 (*w*/*v*) ratio with an ice-cold lysis buffer (containing protease inhibitors). Homogenization was carried out using a mechanical homogenizer (Wisetis HG-15A, Daihan Scientific Co. Ltd., Wonju, Republic of Korea) at 13,500 rpm for two cycles of 30 s, with 1 min intervals on ice to prevent thermal degradation of the enzymes. For the muscle tissue, owing to its dense fibrous structure and high collagen content, a more vigorous approach was employed. The muscle samples were finely minced with sterile surgical scissors before homogenization. The tissue-to-buffer ratio was maintained at 1:10 (*w*/*v*), and the homogenization was performed at a higher speed of 18,000–20,000 rpm for three 30 s bursts to ensure complete cellular disruption. The resulting homogenates were then centrifuged at 10,000× *g* for 15 min at 4 °C. The clear supernatants were carefully collected, aliquoted, and stored at −80 °C for subsequent measurement of inflammatory mediators and oxidative stress biomarkers.

#### 3.8.3. Biochemical Analysis of Serum, Liver, and Muscle Homogenates

The serum and tissue samples were thawed under controlled conditions and processed in accordance with the Enzyme-Linked Immunosorbent Assay (ELISA) protocols provided by the manufacturers. The levels of target biochemical parameters, including MDA, GSH, SOD were quantified using rat-specific ELISA kits (Cat. No: YLA0029RA, Lot No: YLMV883; Cat. No: YLA0121RA, Lot No: YLZ98934 and Cat. No: YLA0115RA, Lot No: YLOMB983; YLBiont, Shanghai, China, respectively), while TNF-α, IL-1β and Insulin were measured using kits from Sunred (Cat. No. DZE201110765, Lot No. 202403, Cat. No: DZE201110120, Lot No. 202403 and Cat. No: DZE201110708, Lot No. 202401, Shanghai, China). All assays were conducted in duplicate following the manufacturers’ instructions. Absorbance was recorded at 450 nm using a microplate reader (Epoch, BioTek Instruments, Winooski, VT, USA). An automatic plate washer (Bio-Tek ELX50, USA) was utilized for efficient washing cycles. The biochemical parameters in serum, liver, and muscle samples were quantified using ELISA kits with a high degree of precision, as evidenced by intra- and inter-assay coefficients of variation (CV) of less than 10%. The measurement ranges and sensitivities (in parentheses) for the target molecules were as follows: for MDA, 20–1600 ng/mL (10.25 ng/mL) for GSH, 0.5–20 ng/mL (0.016 ng/mL), for SOD, 0.2–60 ng/mL (0.113 ng/mL), for COX-1, 0.05–20 ng/mL (0.026 ng/mL), for COX-2, 0.05–10 nmol/mL (0.024 nmol/mL), for TNF-α, 25–8000 pg/L (20.118 pg/L), for IL-1β 8–1000 ng/L (5.127 ng/L, and for Insulin 0.31–20 ng/L (0.19 ng/L). Total protein levels were determined according to the literature and expressed as mg/protein [55,56]. Final tissue concentrations were normalized to the total protein content, accounting for dilution factors and tissue-specific protein ratios. Results were expressed as nmol/mg protein for MDA, ng/mg protein for GSH, SOD, ng/g protein for TNF-α, and pg/g protein for IL-1β. All analyses were conducted at the Kafkas University, Faculty of Medicine, Medical Biochemistry R&D laboratory.

Routine biochemical parameters were quantified using an AU480 automated chemistry analyzer (Beckman Coulter, Brea, CA, USA). Serum urea, creatinine, total cholesterol, HDL-cholesterol, LDL-cholesterol, and triglyceride levels were determined using standard enzymatic-colorimetric methods. The activities of aspartate aminotransferase (AST), alanine aminotransferase (ALT) were measured via kinetic photometric assays in accordance with the International Federation of Clinical Chemistry (IFCC) recommendations. Concentrations were reported in milligrams per deciliter (mg/dL).

### 3.9. Molecular Analyses

In our study, TNF-α, Caspase 3, Caspase 9 mRNA expression levels were compared between groups.

### 3.10. RNA Extraction

The tissues were homogenized in the Tissue Lyser II (Qiagen, Venlo, The Netherlands) and RNA was extracted in the QIAcube. Total RNA isolation steps were carried out on the Qiaqube RNA isolation device (Qiagen, Venlo, The Netherland) using the RNeasy Mini Kit (Qiagen) as recommended by the manufacturer. Total mRNA amount was measured by NanoDrop spectrophotometry (EPOCH Take3 Plate, Biotek) at 260 nm [57].

### 3.11. cDNA Synthesis

cDNA was synthesized from total RNA using the High-Capacity cDNA Reverse Transcription Kit. Each reaction was carried out with 10 μL RNA. cDNA synthesis was achieved with Thermal Cycler (Applied Biosystem, Waltham, MA, USA). The amount of cDNA was determined by NanoDrop spectrophotometry (EPOCH Take3 Plate, Biotek) and stored at −20 °C.

### 3.12. RT-PCR Analyses

mRNA expression of genes was quantified using the Taq Man Gene Expression Master Mix kit. TaqMan Gene Expression Assays JN174232 for rat Caspase-3, JN174233 for rat Caspase-9, JN157530 for rat TNF-α and JN174235 for rat β-actin (Primer Design). Amplification and quantification were performed on the StepOne Plus Real-Time PCR System (Applied Biosystems, Waltham, MA, USA). TaqMan^®^ Gene Expression Assays, suitable for 100 ng cDNA, were pipetted and run for 40 cycles. Ct values were automatically converted to delta delta Ct on the device and analyzed [58].

### 3.13. Histopathological and Immunohistochemical Analysis Procedures

Tissues collected at the end of the experiment were fixed in a 3.7% formaldehyde solution for 72 h. After fixation, the samples were washed under running water, dehydrated using a series of alcohol solutions of increasing concentrations (from 50% to 99%—1 h), and cleared in xylene baths. Subsequently, blocks were prepared from the tissues embedded in paraffin at 60 °C. Sections 5 micrometers thick, obtained using a microtome, were mounted on polylysine-coated adhesive slides.

### 3.14. Hematoxylin and Eosin (H&E) Staining

After being incubated in an oven at 65 °C, the slides were deparaffinized and rehydrated using xylene and a series of decreasing alcohol concentrations. For nuclear staining, the sections were treated with Harris hematoxylin (5 min); for cytoplasmic staining, the tissues were incubated in an Eosin Y solution for 2 min, then re-dehydrated, cleared, and mounted with a coverslip [59].

### 3.15. Immunohistochemical (IHC) Staining

Muscle and liver tissue sections were boiled in sodium citrate buffer under high pressure to expose antigenic sites. Endogenous peroxidase activity was blocked with 3% hydrogen peroxide (H_2_O_2_), and nonspecific background staining was prevented with an appropriate protein-blocking solution. The sections were incubated overnight at +4 °C with primary antibodies (INR, IRS1, GLUT4, NRF2). The following day, after washing steps, secondary antibody and streptavidin-peroxidase applications were performed. The reaction was visualized using the DAB (3,3′-diaminobenzidine) chromogen, and the slides were mounted after completing the background staining with Mayer’s hematoxylin. Negative control staining shared as supplementary files (Figures S1 and S2).

The resulting immunopositivity findings were scored semi-quantitatively as follows, taking into account staining intensity and cell percentage: 0: Immunonegative (0–10% staining), 1: Mild positivity (10–33% staining), 2: Moderate positivity (33–66% staining), 3: Severe positivity (66–99% staining).

### 3.16. Microscopic Evaluation

All prepared H&E and IHC slides were examined and documented using an Olym-pus BX43 microscope (Olympus, Hachioji, Japan) equipped with an integrated camera system. The microscopic images obtained were edited using Adobe Photoshop CS5 software to enable comparative visual analyses between groups.

### 3.17. Statistical Analyses

The data obtained from our study were analyzed using IBM SPSS 20.00 and GraphPad Prism 5.00 software packages. Following the Shapiro–Wilk test, it was determined that the data followed a normal distribution. The Levene’s statistic also confirmed that the variances were homogeneous. Therefore, parametric tests were used. The one-way ANOVA (a parametric test) and the post hoc Tukey test were used to assess statistical significance between groups. A *p*-value of <0.05 was considered statistically significant.

### 3.18. Limitations

As there were no previously published in vivo experimental studies on M. floribunda, the dosage in our study was determined based on data from our previous laboratory experiments. Due to budget constraints, extracts obtained from different solvents could not be tested and detailed mechanistic and molecular analyses could not be performed. MDA was quantified using a competitive immunoassay that detects MDA–protein adducts rather than by the more specific reference methods (HPLC/LC-MS/MS, or the conventional TBARS assay), while SOD and GSH were determined as analyte levels by commercial ELISA kits rather than as enzymatic activities, owing to time and budgetary constraints. The antioxidant findings should therefore be interpreted as reflecting analyte abundance rather than enzymatic function and may not be directly comparable with activity-based or chromatographic data. For reproducibility, these reference methods, particularly enzymatic activity assays, should not be overlooked in future studies. As conducting the study in vivo in rats does not directly demonstrate clinical utility, further preclinical and clinical research is required.

While qPCR efficiently reflects transcriptional dynamics, mRNA expression levels may not always directly correlate with functional protein levels due to post-translational modifications. Although IHC successfully demonstrated cellular localization, it is inherently semi-quantitative and subject to inter-observer variability and fixation artifacts. Lastly, potential cross-reactivity and the detection limits of the commercial ELISA kits should be considered when interpreting absolute protein concentrations.

## Figures and Tables

**Table 1:** NRF2 immunohistochemical scoring in muscle and liver tissue.

Groups	Muscle	Liver
	NRF 2	NRF-2
C	3	3
DM	1	1
DM + MET	3	3
DM + MF 250	2	2
DM + MF 500	2	3

C: Healthy, DM: Diabetes group, MET: Metformin, MF: *Malus floribunda*.

**Table 2:** Immunohistochemical scoring of INR, IRS1, and GLUT4 in skeletal muscle.

Groups	Muscle		
	InR	IRS1	GLUT4
C	3	3	3
DM	1	1	1
DM + MET	3	3	3
DM + MF 250	2	2	2
DM + MF 500	2	3	3

C: Healthy, DM: Diabetes group, MET: Metformin, MF: *Malus floribunda*.

**Table 3:** Quantitative determination of phytochemicals in MF extract.

Compound	mg/g Extract
Shikimic acid	0.033
Gallic acid	0.003
Protocatechuic acid	0.017
Catechin	0.285
Chlorogenic acid	0.340
Caffeic acid	0.004
Vanillin	0.003
p-coumaric acid	0.004
trans-ferulic acid	0.006
Protocatehuic ethyl ester	0.001
Rutin	0.072
Isoquercitrin	0.476
Quercetin-3-D-xyloside	0.161
Kaempferol-3-glucoside	0.001
Quercetin	0.046

## Data Availability

The data presented in this study are available on request from the corresponding author. (The data are not publicly available due to privacy or ethical restrictions).

## Supplementary Materials

The following supporting information can be downloaded at: https://www.mdpi.com/article/10.3390/ijms27125520/s1.
