# Supplementary material for: Malus floribunda Siebold ex Van Houtte Fruit Extract Mitigates Fructose/Streptozotocin Induced Type 2 Diabetes in Rats
Source: Int J Mol Sci. 2026 Jun 18;27(12):5520. doi: 10.3390/ijms27125520 (PMC13299866; doi:10.3390/ijms27125520)

Table S1. Analytical method validation parameters that belong to the LC-ESI-MS/MS

No	Analytes	Ion Source	RT[a]	Ion Transitions	Ion. mode	[b]R^2	[c]LOQ (ug/L)	[d]LOD (ug/L)	Linearity Range (ug/L)
1	Gallic acid	ESI	3.218	169.0 -> 125.1	Negative	0.9986	185.862	71.674	31.25-500
2	Protocatechuic acid	ESI	5.449	153.0 -> 109.0	Negative	0.9969	131.729	31.564	15.625-250
3	Epigallocatechin	ESI	6.796	307.0 -> 139.0	Positive	0.9995	38.750	20.903	12.5-200
4	Catechin	ESI	6.904	288.9 -> 245.1	Negative	0.9946	75.013	17.055	343.750-5500
5	Chlorogenic acid	ESI	7.378	353.0 -> 191.0	Negative	0.9981	259.023	115.890	31.25-500
6	Hydroxybenzaldeyde	ESI	7.679	121.0 -> 92.0	Negative	0.9993	128.651	49.742	15.625-250
7	Vanillic acid	ESI	7.791	167.0 -> 151.8	Negative	0.9958	14.242.132	2.190.421	1250-20000
8	Caffeic Acid	ESI	7.847	178.9 -> 135.1	Negative	0.9994	241.620	69.205	31.25-500
9	Syringic acid	ESI	8.401	197.1 -> 181.8	Negative	0.9990	8.573.388	3.585.000	1250-20000
10	Caffeine	ESI	8.431	195.0 -> 137.9	Positive	0.9986	154.959	68.099	18.75-300
11	Vanillin	ESI	8.649	153.0 -> 125.0	Positive	0.9949	405.411	145.885	62.5-1000
12	o-coumaric acid	ESI	9.441	163.0 -> 119.1	Negative	0.9996	79.973	40.164	15.625-500
13	Salicylic acid	ESI	9.539	137.0 -> 93.1	Negative	0.9981	829.646	476.695	112.5-1800
14	Taxifolin	ESI	9.743	304.8 -> 258.9	Positive	0.9938	235.110	110.294	37.5-600
15	Resveratrol	ESI	9.791	229.0 -> 107.0	Positive	0.9910	135.575	45.806	18.75-300
16	Polydatine	ESI	9.807	390.9 -> 228.9	Positive	0.9987	18.411	11.471	7.8125-125
17	trans-ferulic acid	ESI	10.132	193.1 -> 133.9	Negative	0.9950	115.276	61.184	31.25-1000
18	Sinapic acid	ESI	10.414	223.1 -> 208.0	Negative	0.9972	49.652	19.437	125-2000
19	Scutellarin	ESI	11.151	462.8 -> 286.8	Positive	0.9978	40.013	31.346	9.375-300
20	p-coumaric acid	ESI	11.502	163.0 -> 119.0	Negative	0.9987	175.416	35.348	31.25-500
21	Coumarin	ESI	11.531	147.1 -> 103.2	Positive	0.9974	207.034	60.077	62.5-1000
22	Protocatechuic ethyl ester	ESI	11.622	181.0 -> 107.9	Negative	0.9996	249.201	145.610	15.625-1000
23	Hesperidin	ESI	11.842	611.0 -> 302.9	Positive	0.9957	176.753	41.396	31.25-500
24	Isoquercitrin	ESI	11.867	464.9 -> 302.8	Positive	0.9982	112.680	99.382	18.75-300
25	Rutin	ESI	12.293	611.0 -> 302.8	Positive	0.9980	2.406.720	595.597	125-2000
26	Quarcetin-3-xyloside	ESI	12.433	432.7 -> 299.5	Negative	0.9900	694.059	187.126	125-2000
27	Kaempferol-3-glucoside	ESI	13.287	448.8 -> 286.9	Positive	0.9997	45.238	11.609	7.8125-125
28	Fisetin	ESI	13.327	287.0 -> 137.0	Positive	0.9954	443.662	108.961	15.625-250
29	Baicalin	ESI	13.653	446.8 -> 270.9	Positive	0.9991	30.988	0.5276	15.625-250
30	Chrysin	ESI	14.23	254.9 -> 153.0	Positive	0.9989	0.1338	0.0737	1.5625-25
31	trans-cinnamic acid	ESI	14.272	149.0 -> 131.1	Positive	0.9999	220.279	111.853	31.25-500
32	Quercetin	ESI	14.821	300.8 -> 151.0	Negative	0.9964	169.127	46.558	27.5-440
33	Naringenin	ESI	14.999	270.9 -> 119.1	Negative	0.9960	0.4575	13.694	31.25-500
34	Hesperetin	ESI	15.815	300.9 -> 164.0	Negative	0.9966	0.6496	0.3008	31.25-500
35	Morin	ESI	15.819	302.8 -> 153.0	Positive	0.9981	0.5284	0.1253	1.5625-50
36	Kaempferol	ESI	16.431	284.9 -> 116.9	Negative	0.9997	54.004	18.683	312.5-10000
37	Baicalein	ESI	17.084	271.0 -> 123.0	Positive	0.9988	0.9631	0.5955	1.5625-25
38	Luteolin	ESI	17.909	285.0 -> 133.1	Positive	0.9962	214.535	200.000	31.25-500
39	Biochanin A	ESI	17.91	284.9 -> 151.9	Positive	0.9963	0.7333	0.1475	1.5625-25

[a]R.T.: Retention time, [b]R^2: Coefficient of Determination, [c]LOQ (ug/L): Limit of quantification, [d]LOD (ug/L): Limit of detection.

Figure S1. Liver Negative Control Staining

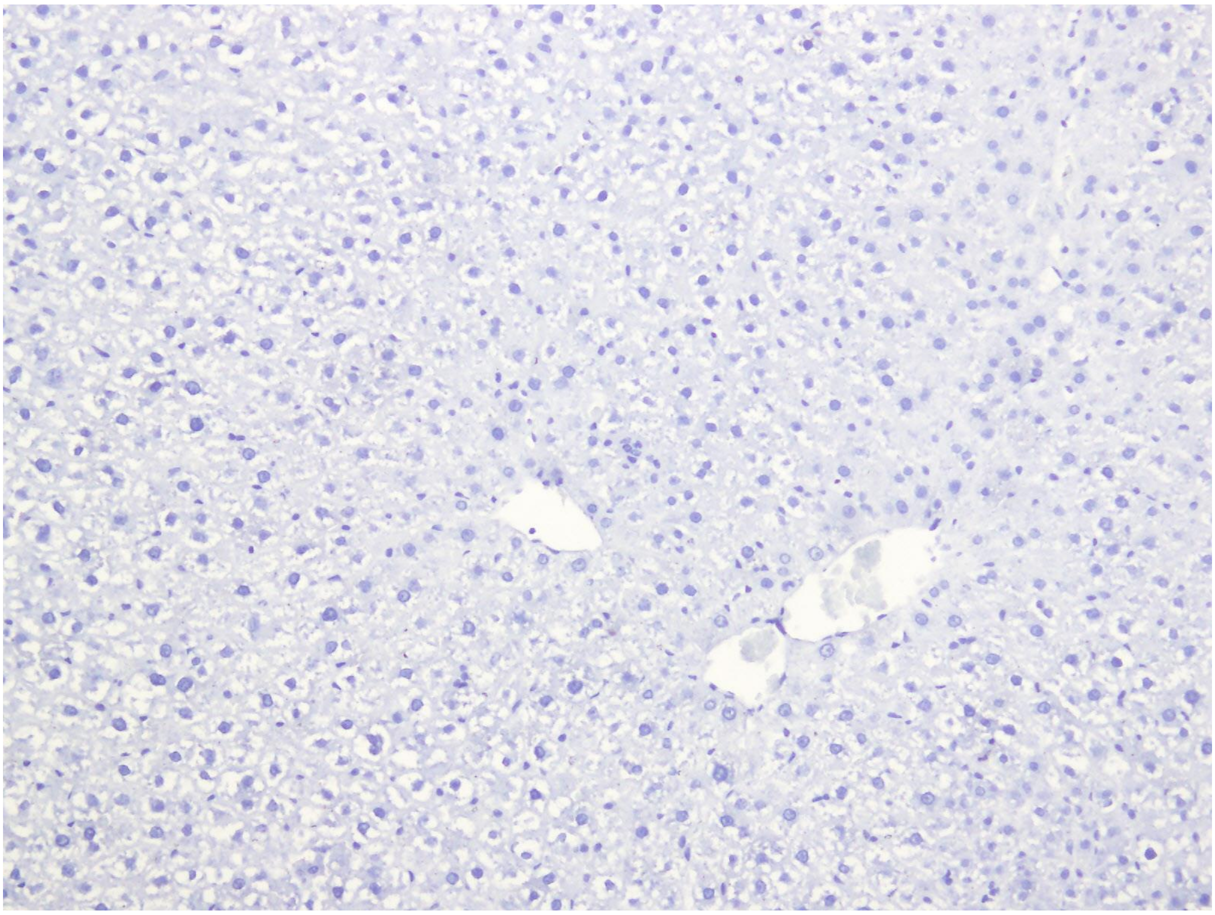

Figure S2. Muscle Tissue Negative Control Staining

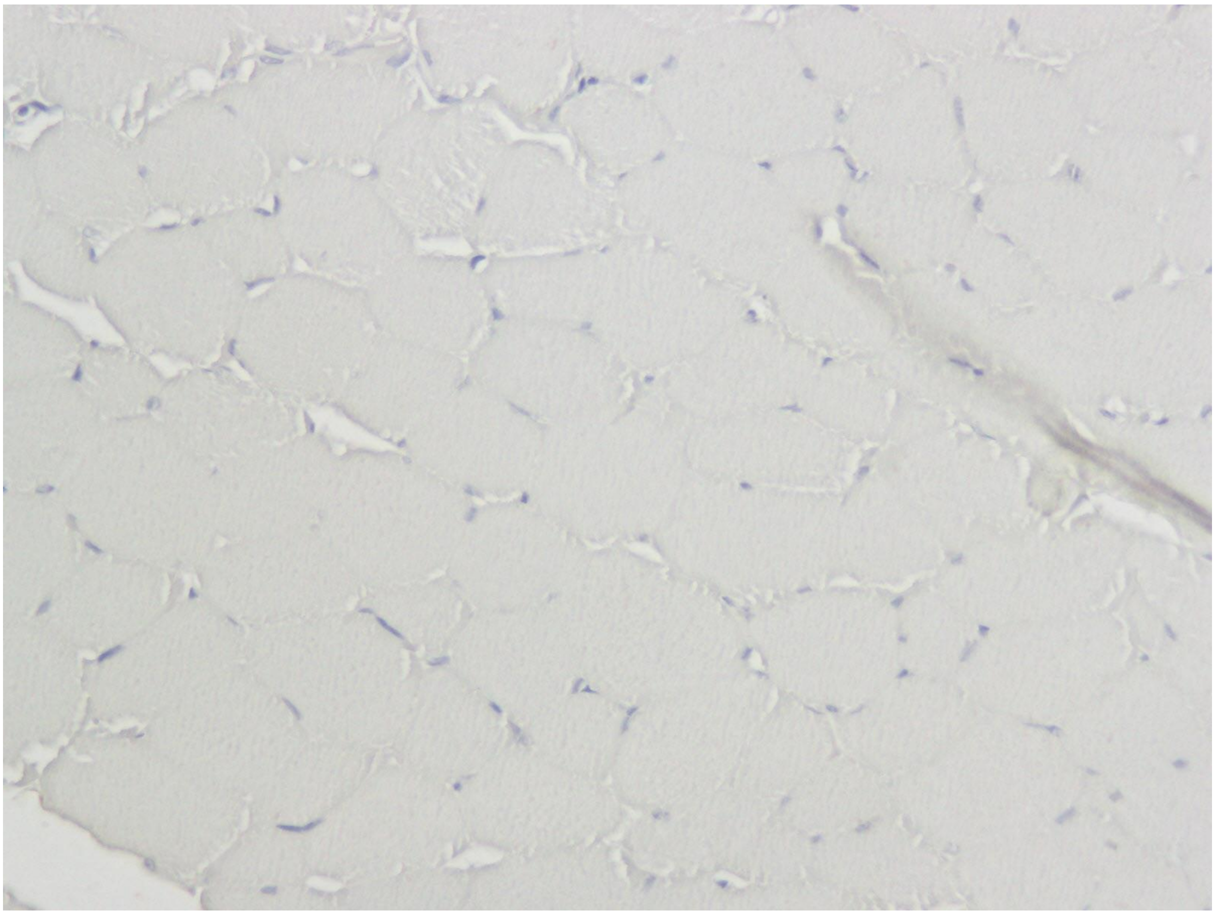

Supplement: Supplementary file 1 [file ijms-27-05520-s001.zip › ijms-4302958-supplementary.pdf]
